# Management and prevention of acute bleedings in the head and neck area with interventional radiology

**DOI:** 10.1186/s13005-016-0103-3

**Published:** 2016-01-23

**Authors:** Katharina Storck, Kornelia Kreiser, Johannes Hauber, Anna-Maria Buchberger, Rainer Staudenmaier, Kilian Kreutzer, Murat Bas

**Affiliations:** Department of Otorhinolaryngology, Klinikum Rechts der Isar, Technische Universität München, Ismaningerstrasse 22, 81675 Muenchen, Germany; Department of Diagnostic and Interventional Neuroradiology, Klinikum Rechts der Isar, Technische Universität München, Ismaningerstrasse 22, 81675 Muenchen, Germany; Department of Maxillofacial Surgery, Universitaetsklinikum Eppendorf, Martinistraße 52, 20246 Hamburg, Germany

**Keywords:** Interventional neuroradiology, Haemorrhage, Embolization, Head and neck, Vascular erosion

## Abstract

**Background:**

The Interventional Neuroradiology is becoming more important in the interdisciplinary treatment of acute haemorrhages due to vascular erosion and vascular tumors in the head and neck area. The authors report on acute extracranial haemorrhage in emergency situations but also on preventive embolization of good vascularized tumors preoperatively and their outcome.

**Methods:**

Retrospective analysis of 52 patients, who underwent an interdisciplinary approach of the ORL Department and the Interventional Neuroradiology over 5 ½ years at the Department of Otorhinolaryngology, Klinikum Rechts der Isar, Technical University of Munich, Germany. Their outcome was analysed in terms of success of the embolization, blood loss, survival rate and treatment failures.

**Results:**

39/52 patients were treated for acute haemorrhage. Twenty-five of them attributable to vascular erosion in case of malignant tumors. Affected vessels were the common carotid artery as well as its internal and external parts with branches like the ascending pharyngeal, the facial and the superior thyroid artery.

Altogether 27/52 patients were treated for malignant tumors, 25/52 were attributable to acute haemorrhage due to epistaxis, after tonsillectomy, benign tumors and bleeding attributable to inflammations. Treatment of all patients consisted either of an unsuccessful approach via exposure, package of the bleeding, electrocoagulation or surgical ligature followed by embolization or the primary treatment via interventional embolization/stenting.

**Conclusions:**

The common monitoring of patients at the ORL and interventional neuroradiology is an important alternative especially in the treatment of severe acute haemorrhage, following vascular erosion in malignant tumors or benign diseases. But also the preoperative embolization of good vascularized tumors must be taken into account to prevent severe blood loss or acute intraoperative bleeding.

## Background

The incidence of the therapeutical use of interventional neuroradiology in the head and neck is increasing especially in acute bleeding situations. The first description of therapeutic percutaneous embolization in intractable epistaxis appeared as early as 1974 [1]. Because of the intimate anatomy of the extracranial and intracranial vascular system, the treatment of acute haemorrhage and also the preoperative treatment of hypervascular tumors by an endovascular or transcutaneous technique is still reserved for specific indications and, in most cases, is still not the first choice of treatment. Classic indications may include acute arterial haemorrhage attributable to vascular erosions in carcinomas, refractory epistaxis or bleeding after tonsillectomy. But also the preoperative “downstaging” of benign and malign tumors represents a considerable amount of cases, in order to prevent severe blood loss or intraoperative haemorrhage. We present a retrospective study of 52 patients who attended the Department of Otolaryngology (ORL) of the Technical University of Munich (TUM) and who underwent intra-arterial embolization treatment at the Department of Interventional Neuroradiology, TUM, during a time span of January 2007 to May 2012 (5 ½ years).

## Methods

### Patients

Data were collected during a 5.5-year period. Charts were reviewed for demographics, range of indications for embolization, tumor localization and size, initial oncological treatment, localization of bleeding, tumor recurrence, survival outcome (Munich cancer register) and failure rates of the embolization. All research has been approved by the authors’ ethic committee of the Technical University of Munich (Permit Number: 251/15). Fifty-two patients underwent interventional procedures including selective angiographic or transcutaneous embolization/stenting. The mean age of the entire cohort was 54.9 ± 19.7 years (Range 11–94 years). Of these patients, 35 were male and 17 were female; 38 patients presented with tumors (*n* = 27 malignant tumors, *n* = 11 benign tumors), nine patients with refractory epistaxis, three patients with refractory bleeding after tonsillectomy and two patients with bleeding because of inflammation.

The mean age of 64.8 ± 11.8 years was the highest in patients with malignant tumors and the lowest with 27.3 ± 4.8 years in patients with bleeding after tonsillectomy. Patients of the ORL, who underwent other intracranial interventions, e.g. attributable to apoplexy, were excluded.

### Embolization

Generally, percutaneous embolization was performed under general anaesthesia after imaging diagnostics such as magnetic resonance imaging (MRI) or computerized tomography (CT)-scan. In every case a guiding catheter (e.g. 6 F Envoy® [Codman]) was positioned supra-aortic in the internal carotid artery (ICA), external carotid artery (ECA), vertebral artery (VA) or branches of the thyrocervical trunk by a transfemoral, transarterial access over a hydrophilic guide wire (e.g. 0.35 Glidewire® [Terumo]). The bleeding or the pathological vessels could be localized by injection of a contrast agent (Imeron 300® [Bracco]) into the vessels. In cases of solid tumorous masses with a subcutaneous or intranasal superficial localization, direct transcutaneous or transnasal respectively puncture was considered; therefor a 20 G, 88 mm coaxial needle (Spinocan® [B.Braun]) was placed under biplane fluoroscopy by means of a transarterial “roadmap”. The correct position was checked by an injection of contrast agent directly through the needle. If the ICA ran nearby the target lesion a balloon was temporarily inflated in the ICA to prevent embolization of the liquid into intracranial vessels (Copernic®, BALT).

In cases of a more diffuse bleeding or multiple rather separated affected vessels the intra-arterial access was the approach of choice, in doing so a microcatheter (Prowler select® [Codman], Nautica™, Rebar™, Echelon™, Marathon™, Apollo™ [ev3]) was delivered to the target vessel over a microwire (syncro^2^standard® [Boston scientific], Traxcess® [Microvention]).

Embolization liquids were synthetic glue (Glubran2, [GEM S.r.l.], Histoacryl® [B. Braun]) diluted with an oily contrast agent (Lipiodol®UltraFluid [Guerbet]) at a ratio of 1:3 or an ethylene vinyl alcohol copolymer dissolved in dimethyl sulfoxide (Onyx®LES [Covidien]). Solid embolization devices were polyvinyl alcohol (PVA) particles of different sizes (150–355, 250–350, 500–700 μm; BeadBlock™ [Terumo]; Contour™ [Boston Scientific]) or bare metal coils (Axium™, Helix™ Coils, [ev3]; Deltaplush™ [Codman]), fibered coils (Helix™Fibered Coils, [ev3]) respectively. In three cases stent grafts (Fluency® [BARD]) were implanted in the common carotid artery, in one of these patients five days later the same vessel was occluded with vascular plugs (Amplatzer® [St.Jude Medical]) proximal and distal of the lesion.

## Results

### Acute versus elective embolization

**Fig. 1:**
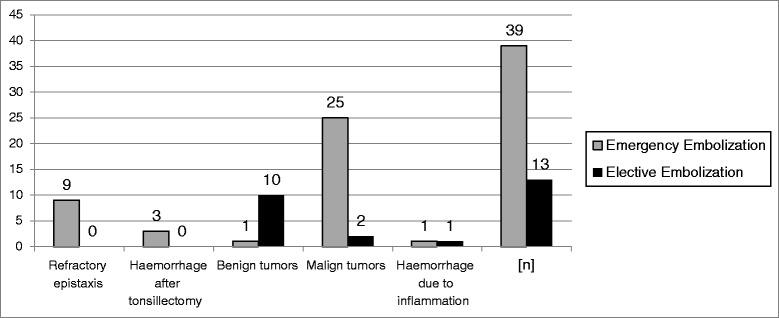
Distribution of diagnosis-related emergency versus elective embolization

39/52 patients (75 %) developed acute arterial haemorrhage. In 25 cases due to vascular erosion in malignant tumors, followed by refractory epistaxis (*n* = 9) and haemorrhage after tonsillectomy (*n* = 3). Only one benign tumor and one haemorrhage due to inflammation led to an acute intervention. Figure 1 shows the distribution of emergency and elective embolization depending on the diagnosis. Especially with respect to the emergency embolization of acute haemorrhage, 34/39 were successful and five unsuccessful in terms of stopping the haemorrhage.

13/52 patients underwent elective intra-arterial embolization mostly to prevent intraoperative severe blood loss due to good vascularized tumors. The elective embolization was successful in 9/13 cases in terms of “downstaging” the vascularization and successful resection of the tumor within 48 h after the embolization and failed in four cases all due to the lack of feeding vessels.

**Fig. 2:**
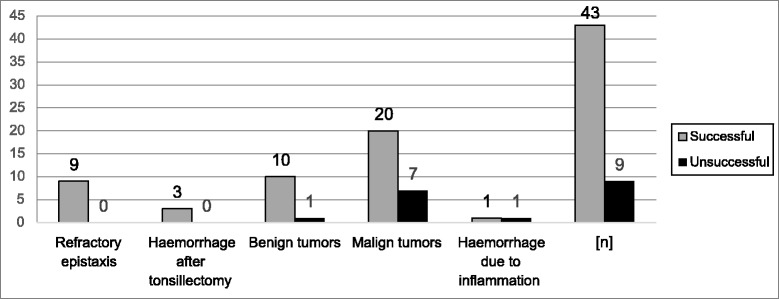
Diagnosis-related success rate of the embolization

Figure 2 shows the success rate based on the diagnosis. One benign tumor resulted in a papilloma without feeding vessels. Seven failures occurred in malignant tumors, of which no feeding vessels could be detected in five cases, a palliative and only uncompleted embolization via direct puncture could be accomplished in one case and no stable embolization position could be found in one case. The last case was an unclear pharyngeal bleeding without feeding vessels with spontaneous suspension.Table 1 shows the reasons for the failure of the embolization.

### Malignant tumors

27/52 patients were treated for malignant tumors, 25/27 of them for acute haemorrhage. Only two patients were treated for the preoperative downstaging of malignant tumors; 21/27 patients presented with head and neck squamous cell carcinoma (HNSCC) classified T1 through T4, three with thyroid carcinoma and three with other malignant tumors. Of the 21 HNSCC, 12 presented oropharyngeally, three hypopharyngeally, three laryngeally, one oro-, hypopharyngeally (2-stage carcinoma) and one oro-, hypopharyngeally and laryngeally (3-stage carcinoma). In four patients, bleeding came from the primary tumor (initial diagnosis). In 18 cases, the bleeding was attributable to a recurrence of the primary tumor (three thyroid carcinomas, 15 HNSCC). Only three patients presented with haemorrhage without histological evidence of a recurrence of the tumor. In two patients the preoperative attempt to downstage the tumor by embolization was used without success, due to missing pathological afferent vessels. Of the 21 HNSCC patients, 13 developed acute arterial haemorrhage after primary radiotherapy, seven after surgery with adjuvant radiotherapy and only one patient after primary surgery. We also analysed the embolized vessels. A total of 36 vessels were embolized/stented: in 31 cases, branches of the external carotid artery (12 times superior and inferior thyroid artery) and in five cases, branches of the subclavian artery.

Due to a tumorous erosion of the common carotid artery a covered fluency stent graft was used for the reconstruction in two cases. Another covered fluency stent graft was used for an erosion of the common carotid artery due to a chronical inflammation.

**Fig. 3:**
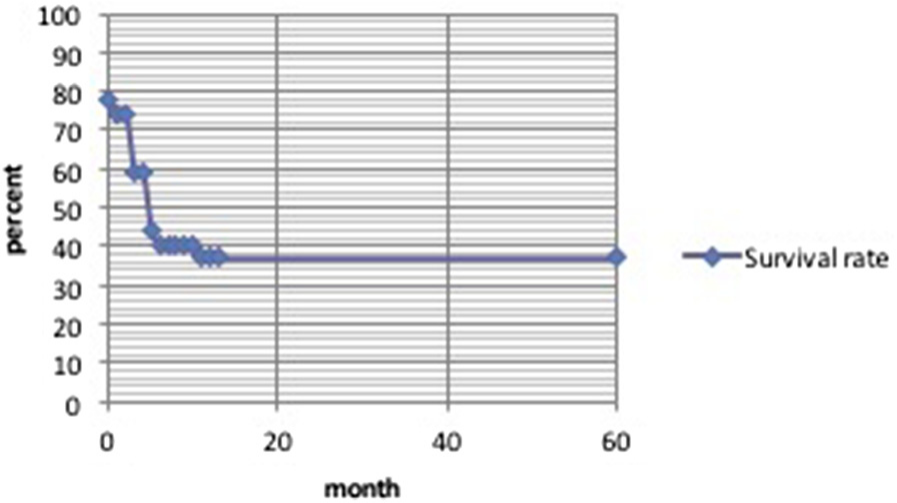
Long-term follow-up of a maxiumum of 60 months shows a overall survival rate of 38 % of all tumor patients

With the assistance of the Munich Cancer Register we also obtained data concerning the survival rate of all tumor patients. The long-term follow-up of a maximum of 60 months post-interventional showed an overall survival rate of 38 % (Fig. 3).

### Benign tumors

Eleven patients were treated for benign tumors preoperatively by embolization in order to minimize the vascularization and the intra-surgery bleeding. 10/11 underwent preoperative embolization, one an acute intervention due to acute intraoperative bleeding. Four patients had tumors of the glomus caroticum. All patients described an increasing not painful swelling of the neck.

**Fig. 4:**
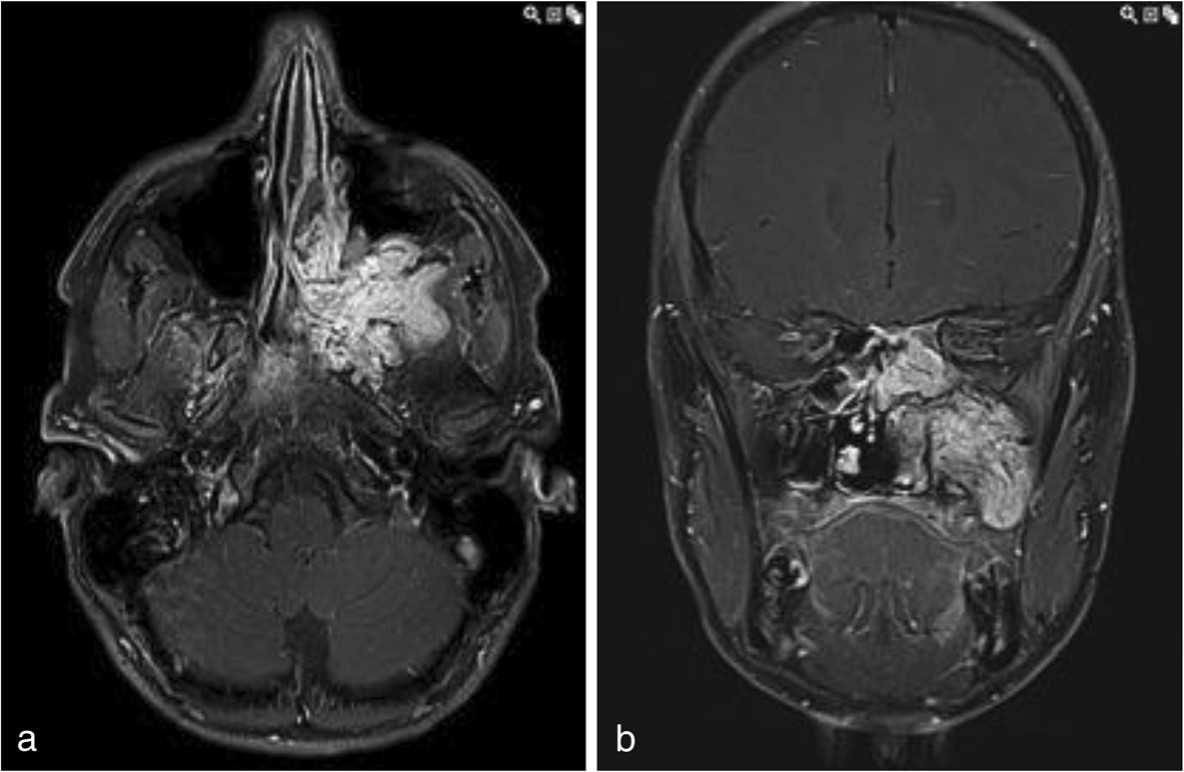
Preoperative MRI Scan of a 17-year-old boy with a juvenile nasopharyngeal angiofibroma: **a** shows the axial view and **b** shows the coronary scan

**Fig. 5:**
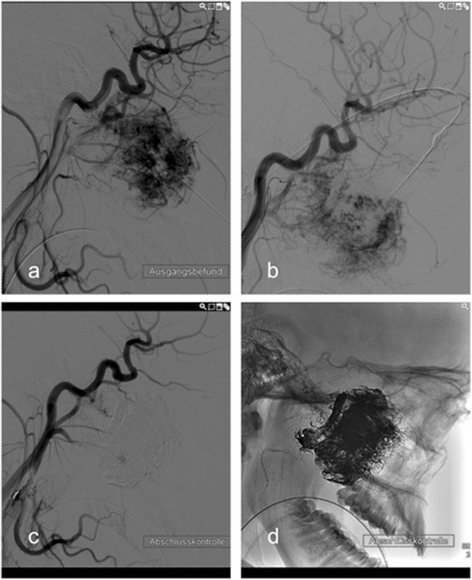
Embolization of the juvenile nasopharyngeal angiofibroma via direct puncture, all in lateral view: **a** pre-interventional digital subtraction angiography. **b** example of the needle position in lateral view. **c** post-interventional control via digital subtraction angiography. **d** post-interventional digital subtraction angiography without image subtraction

Three young male patients with juvenile nasopharyngeal angiofibromas describing the typical symptoms as nose blockade, swelling of the face, headache or epistaxis. In all three patients the preoperative embolization of the tumors resulted in a satisfying resectability of the nasopharyngeal angiofibroma via midfacial degloving without remarkable side effects. Within the follow-up time span of this research none of these young patients presented with the recurrence of the tumor. Two patients presented with haemangioma with a progredient swelling of the temporal or submandibular region and one each of papilloma and chondroma. In Fig. 4, the preoperative MRI scans of a 17-year-old boy with a juvenile nasopharyngeal angiofibroma are shown. Figure 5 shows the embolization via direct puncture of the tumor.

### Epistaxis

All patients (*n* = 9) who underwent emergency intra-arterial embolization for epistaxis were first treated conservatively via nasal packing and/or cautery under local anaesthesia and local vasoconstrictors or under general anaesthesia via endoscopic surgery or by septumplasty, all without success. In eight cases, posterior epistaxis could be seen. In one case, an anterior/ posterior epistaxis in a patient with Osler’s disease could be detected. In seven cases, the sphenopalatine artery was embolized, in one case the maxillary artery, in one case the ascending palatine artery and in one case the descending palatine artery. Three of the patients suffered from hypertension, two of them being additionally treated with anticoagulants. One patient suffered from Osler’s disease and is well known in our department, since he presents with epistaxis at frequent intervals.

### Haemorrhage after tonsillectomy

Three patients presented with severe haemorrhage after tonsillectomy. None of them were operated upon in our department. All three cases were refractory to the primary attempt of operative haemostasis through the oral cavity under general anaesthesia via ligation, cautery and oral packing. As in all three cases the colleagues of the interventional neuroradiology were already prepared for a possible intervention, embolization of the arteries was performed immediately after the operation, instead of ligating the external carotid artery via a cervical approach. The facial artery was embolized with success and neck surgery to prepare and ligate the carotid artery or it’s branches was not necessary.

### Blood loss

#### Malignant tumors

We additionally analysed the blood loss as it is one of the main reasons for the intervention to be considered. Concerning the malignant tumors, the blood loss due to acute haemorrhage was high. Of 27 patients, 15 were unsuccessfully treated by surgical haemostasis primarily. In three patients, no blood transfusion was needed. In these cases the preoperative haemoglobin level was 11.0 (±1.5) [g/dL] and 10.1 (±2.0) [g/dL] after the unsuccessful surgery. The preoperative haemoglobin level of the other twelve patients was 9.0 (±2.5) [g/dL] and postoperatively 9.2 (±2.3) [g/dL]. These twelve patients all together received 16 fresh frozen plasma and 74 red cell concentrates. 12 patients were treated by primary embolization without a primary surgical intent due to the dramatic bleeding. 10/14 did not receive blood transfusion since the haemoglobin level before the embolization was 12.2 (±1.9) [g/dL] and after the procedure 10.9 (±2.0) [g/dL]. In two patients, a blood transfusion was needed. The haemoglobin level was 9.8 (±1.0) [g/dL] before and 11.1 (±2.3) [g/dL] after the embolization. These two patients received twelve red cell concentrates all together.

#### Benign tumors

Especially in the preventive preoperative embolization of good vascularized benign tumors, the minimization of the intraoperative blood loss and the occurrence of an acute intra- or postoperative bleeding was a major issue of the embolization. One was primarily treated surgically and afterwards by embolization due to acute intraoperative bleeding. In this case, the preoperative haemoglobin level was 15 [g/dL] and after the operation 12.2 [g/dL]. Before embolization, we measured a value of 12.2 [g/dL] and afterwards a value of 9.4 [g/dL]. Hence, blood loss in total was 5.6 [g/dL]. 10/11 benign tumors received a preoperative embolization of the tumor. In eight cases, no blood transfusion was needed. The haemoglobin level before the embolization was 13.4 (±1.1) [g/dL] and after the embolization 12.3 (±1.9) [g/dL]. Surgical procedures were then performed within 48 h. The haemoglobin level was then preoperatively 14.3 (±0.8) [g/dL] and after the surgery 13.4 (±1.1) [g/dL]. In two cases, blood transfusion intraoperatively was necessary (one tumor of the glomus caroticum and one juvenile nasopharyngeal angiofibroma). Here, the haemoglobin level was 13.0 [g/dL] and after the embolization 12.1 [g/dL]. Before surgery, the haemoglobin level was 11.4 [g/dL] and after surgery and three erythrocyte concentrates 8.1 [g/dL].

One special case that needs to be mentioned was a young girl following the ingestion of an alkali liquid in an attempt of suicide and a subsequent infection of the neck due to recurrent fistulas. A severe haemorrhage of the common carotid artery attributable to inflammation occurred with dramatic blood loss. The preoperative haemoglobin level was 9.1 [g/dL] and postoperative 6.5 [g/dL] after 20 RCCs (red cell concentrates) and 20 packed blood plasma.

The Table 2 shows the total need of RBCs and blood plasma.

**Table 1:** Reasons for the failure of embolization

Dignity	Diagnosis	Reason for failure
Benign tumor	Papilloma of the soft palate	No feeding vessel
Malign tumor	Hypopharyngeal HNSCC	Palliative and incomplete embolization via direct puncture
	CUP-Syndrom	No feeding vessel
	Oropharyngeal HNSCC	No feeding vessel
	Oropharyngeal HNSCC	No feeding vessel
	Plasmozytoma of the sphenoid sinus	No feeding vessel
	Oropharyngeal HNSCC	No feeding vessel
	Oropharyngeal HNSCC	No stable embolization position
Hemorrhage due to inflammation		No feeding vessel with spontaneous suspension of the bleeding

**Table 2:** Diagnosis-related need of RBCs and blood plasma

	Total number of patients [n] = 52	Number of patients with transfusions [n] = 21	Amount of RCC’s [n] = 120	Amount of fresh frozen plasma [n] = 32
Epistaxis	9	3	8	2
Haemorrhage after tonsillectomy	3	2	3	0
Benign tumors	11	2	3	0
Malign tumors	27	14	86	16
Haemorrhage attributable to inflammation	2	1	20	14

### Selective angiography and embolization versus direct puncture

In 52 patients, 44 received selective angiography and embolization or stenting due to active haemorrhage. Only 8 % were treated by direct puncture of the tumor. All of them were elective preoperative procedures (6 benign tumors, 2 malign tumors) for the minimization of the vasculature. Table 3 shows the distribution between the two strategies relating to the diagnosis.

**Table 3:** Direct puncture versus selective angiography and embolization

	Direct puncture	Selective angiography and embolization
Epistaxis	0	9
Haemorrhage after tonsillectomy	0	3
Benign tumors	6	5
Malign tumors	2	25
Haemorrhage attributable to inflammation	0	2

Since various materials for the embolization or stenting of vessels are available, we also determined the frequencies of these possibilities. In total, materials were used in 45 cases. In seven cases, bare metal coils were used, in nine cases a mixture of acrylic glue (Glubran2®:Lipidol (1:3)), in one case ethylene vinyl alcohol (Onyx®) and in three cases fibre coils. To ensure the patency of the carotids and to seal the outgoing vessels, covered stentgrafts (Fluency®) were used in two patients. In all other cases (*n* = 23), combinations of the above-mentioned materials were employed. No statistical significance was seen between the different materials, the outcome concerning the haemorrhage and the complications and side effects.

## Discussion

In situations of acute haemorrhage and the preoperative management of vascular tumors in the extracranial head and neck area, an interdisciplinary approach of head and neck surgeons and interventional neuroradiologists sometimes offers the best therapy options. However, the selection of those patients who are suitable for this approach requires deep considerations of the pros and cons concerning their risk profile. The insertion of a microcatheter into the tumor or haemorrhage feeding vessels and the following embolization via various materials under general anaesthesia requires wide experience and a profound knowledge of the anatomy. In particular, anastomoses between the extracranial and intracranial circulations are potentially dangerous and, hence, a thorough knowledge of anatomy is essential to minimize the risk of cranial nerve palsies, blindness or stroke to name just a few. Liquid agents and small particles of < 150 μm should be used with caution because of the previously mentioned risks [2]. Our aim was to provide a broad analysis of all the data from 52 patients who underwent interventional procedures as an interdisciplinary approach between the ORL and Department of Interventional Neuroradiology of the “Klinikum Rechts der Isar”, Technical University of Munich, during a 5.5-year period. The data obtained in this analysis comprises a rather heterogeneous patient population. The same applies to the choice of embolization material and interventional radiological techniques used. Taking into account that situations of acute haemorrhages are predominant with 75 % a continuous and recurring workflow could not be abstracted from the medical records. Unfortunately, this study is addressing a question which is for clinical an ethical reasons not transferable in a blinded and controlled trail and so has to deal with the limitations of retrospective gained data. To our knowledge there is no comparable data published so far.

The indications for interventional radiology can be divided into three categories [3]. The first category includes acute haemorrhage such as acute carotideal bleeding and also epistaxis. The carotid blowout can be defined as “bleeding from the carotid artery or its branches” and is one of the most feared complications, especially in advanced head and neck malignancy, whether treated surgically or with radiotherapy [4]. In our cohort, three patients suffered from tumors or inflammatory diseases which spreaded straight to the carotid arteries. The second category includes vascular lesions (e.g. tumors such as haemangioma, paraganglioma or juvenile nasopharyngeal angiofibroma or arteriovenous malformations and fistulas). The third category includes venous sampling for parathyroid hormones in the diagnostics of primary hyperparathyroidism, which is not considered in this paper [3]. Another field is the intra-arterial chemo-embolization of oral and oropharyngeal cancer, which is also not considered in this study because of the lack of patients treated by this option [5].

In our cohort nine patients (17 %) were treated for refractory epistaxis by embolization. Compared with an average of 669 (±24.8) patients a year presenting to our service with moderate to severe epistaxis and 31 of them who needed treatment under general anesthesia, the total number of nine patients in 5.5 years is small. We think it is a good alternative especially in posterior epistaxis but as shown by the results it is reserved to special refractory bleeding which can not be stopped even by an endonasal surgical approach circumventing an extracranial approach. We have not seen any of the complications described in the literature such as apoplexy, facial nerve paresis, blindness or haematomas [6]. However, a total amount of nine is too small for a reliable statement to be made. Based on long-term follow-up, we had to exclude one patient with Osler’s disease who repeatedly presents to us with epistaxis. Nevertheless, the interventional embolization in the case of refractory epistaxis can be a secure and effective alternative [7–9].

Consistent with the literature, the occurrence of refractory bleeding after tonsillectomy needing to be treated by embolization is extremely low [10]. From an average of 79 (±19.53) patients per year presenting with haemorrhage after tonsillectomy and 32 of them treated under general anaesthesia, the total of three patients in 5.5 years is gladly very low. It is a good minimal invasive alternative to the conventional ligation of the afferent carotideal branches by open neck surgery if available.

In benign vascular tumors in particular, the preoperative embolization of the feeding vessels might be a good option to minimize the risk of intraoperative bleeding and to increase the possibility of resecting the whole tumor. The embolization of nasopharyngeal angiofibromas and other benign tumors has previously been described in the literature [11, 12]. In the case of juvenile angiofibromas, preoperative embolization might minimize intraoperative bleeding. In such cases, the operation site for endoscopic resection is clearer and the possibility for resecting the whole tumor and minimizing the risk of recurrence is higher. A comparison of patients with angiofibromas with or without preoperative embolization in 1975 showed that, in cases of preoperative embolization, the blood loss was halved [13].

The study of Tang et al. showed a medium range of 1 ½ litres of blood loss intraoperatively after embolization in 13 patients and only 500 ml in cases of transnasal resection. The average range of blood reserve usage was 4 l [14]. Similar results have been shown for paragangliomas and vascular malformations [15, 16]. Since, in our department, all patients with the mentioned tumors are embolized before the operation, we cannot compare the blood loss with or without the preoperative embolization, but as seen in our results only two patient needed in total three red blood cell concentrates during surgery.

The largest number of interventions involved haemorrhage attributable to malignant tumors. Of 27 patients, eight women and 19 men underwent intra-arterial or transcutaneous embolization for oncological reasons. Consistent with the known distribution of malignomas in the head and neck area, 21 patients had HNSCCs [17]. Concerning the 25 patients with haemorrhage, 21 only developed acute arterial bleeding after specific time intervals following primary therapy. Of these patients 18 patients revealed evidence of recurrent tumor disease as established by biopsy and imaging techniques (14 after previous primary R(C)T and only four after previous surgery and adjuvant R(C)T). Only three patients did not present a recurrent tumor. In comparison with these data, Greve et al. found no recurrence of tumor in all presented 10 cases [18].

In our patients, haemorrhage in malignant tumors had two different reasons. On the one hand, the recurrence of the tumor in many cases might cause an infiltration of the vessels with leakage and haemorrhage. On the other hand, bleeding is caused by long-term effects of irradiation on the vessels such as arteriosclerosis and necrotizing vasculitis [19]. These late irradiation effects might be attributable to chronic oxidative stress [20]. Especially after primary R(C)T due to inoperable tumors the doses of the radiotherapy is higher.

In four cases, haemorrhage occurred as early as the primary diagnosis of cancer. In these cases, the tumor had already infiltrated the surrounding vessels causing the bleeding.

In the long-term follow-up at 60 months, the survival rate was still 38 %, despite the high rate of recurrent tumors and the inoperability of most of the tumors. So we can assume that the embolization/stenting of the acute haemorrhage in malignant tumors can prolong the survival rate in some cases where the conventional surgery has its limits. Regarding blood loss, patients with malignant tumors and primary unsuccessful surgery had the highest need of red blood cell concentrates, with 74 RBC and 16 packs fresh frozen plasma in only twelve patients.

## Conclusion

Interventional radiology has an increasingly important position in the treatment especially of acute haemorrhages but also vascular tumors in the head and neck area. In the treatment of acute bleeding due to benign diseases it minimizes the necessity of large incisions and in malignant tumors it sometimes reveals a good treatment alternative in chronical altered soft tissue due to radiotherapy or surgery. The common monitoring of these patients at the ORL, the anaesthesiologists and the Department of Interventional Radiology is necessary, especially in the treatment of severe haemorrhage following not only vascular erosion in malignant tumors, but also benign diseases such as epistaxis. However, the indication for embolization or stenting of the tumor or feeding vessels must be considered carefully because of specific complications.
